# Experimental Evaluation of Rolling Resistance in Omnidirectional Wheels Under Quasi-Static Conditions

**DOI:** 10.3390/s25165026

**Published:** 2025-08-13

**Authors:** Sławomir Duda, Grzegorz Gembalczyk, Tomasz Machoczek, Zygmunt Kowalik

**Affiliations:** 1 Department of Theoretical and Applied Mechanics, Silesian University of Technology, Konarskiego 18A, 44-100 Gliwice, Poland; slawomir.duda@polsl.pl (S.D.); tomasz.machoczek@polsl.pl (T.M.); 2 Department of Mechatronics, Silesian University of Technology, Akademicka 2A, 44-100 Gliwice, Poland; zygmunt.kowalik@polsl.pl

**Keywords:** omnidirectional wheels, rolling resistance, experimental mechanics, quasi-static testing, wheel–surface interaction, force measurements, mobile robotics

## Abstract

This paper presents the results of experimental research on rolling resistance forces occurring during the motion of omnidirectional wheels equipped with dual rows of passive rollers. Due to the complexity of wheel–surface interactions and the stochastic nature of contact transitions, such wheels are often characterized experimentally rather than analytically. A custom-built test stand was used to measure resistance forces for different wheel orientations (0°, 30°, 45°, 60°, and 90°) and two vertical loads (117.7 N and 215.8 N) on two surface types: industrial concrete and anodized aluminum. The results demonstrated a strong influence of wheel orientation on resistance, with the highest mean force recorded at 60° for both loads. The results revealed an oscillatory pattern in the resistance force, strongly influenced by the angular position of the wheel. For concrete, mean forces ranged from 1.04 N to 10.34 N, while for aluminum, they ranged from 1.08 N to 10.11 N. Significant oscillations and occasional negative force values were observed, attributed to roller geometry and wheel irregularities. The data obtained are useful for validating numerical models and improving the design and control of mobile robots using omnidirectional wheels.

## 1. Introduction

The rolling resistance of wheels in mechanical vehicles is one of the most critical parameters influencing the energy consumption of wheeled vehicles. This applies to both combustion-engine vehicles and electric vehicles, including mobile robots [1]. Minimizing rolling resistance is particularly relevant for such vehicles, as they typically operate on flat surfaces at relatively low speeds. As a result, the impact of gradient resistance and inertial forces is less significant over extended driving cycles. Rolling resistance arises from the deformation of the contact surfaces between wheels and the ground, internal friction in the tire structure, and adhesive forces between the tire and the road surface [2]. The magnitude of rolling resistance primarily depends on the type of surface, tire characteristics, and the vehicle’s mass. The higher the rolling resistance, the more energy is required to overcome it [3].

The literature features numerous studies describing methods for determining the rolling resistance of automobile tires [4,5,6,7]. This is due to the widespread use of vehicles equipped with tires and the manufacturers’ aim to achieve an optimal balance between energy efficiency and other tire parameters, such as grip, wear resistance, and more. In general, methods for measuring rolling resistance can be divided into two categories: tests conducted under real road conditions and those performed on specially designed laboratory test stands.

One of the most commonly used methods for determining wheel rolling resistance is the so-called trailer method. This approach uses a trailer equipped with one or more test wheels pulled by a vehicle, and the rolling resistance of the test wheels is measured using force transducers [8]. It is employed in both field and laboratory studies. Each approach has its advantages and disadvantages. Field tests are susceptible to many disturbing factors and heavily depend on weather conditions, making it difficult to ensure repeatable measurement conditions for large samples or repeated trials, especially compared to laboratory methods.

Laboratory testing of tire rolling resistance offers the advantage of controlled testing conditions (including ambient temperature and humidity) and high repeatability of results due to the absence of random disturbances typical of field tests. Laboratory test stands are generally categorized into two groups: those with flat belts and those with cylindrical drums. The methodology for measuring rolling resistance is typically straightforward. In laboratory conditions, it involves pulling a main frame to which a test wheel is mounted. This frame is usually pulled using a cable, one end of which is attached to the frame, while the other is wound onto a drum driven by an electric motor. The force required to move the frame is measured using a force sensor integrated into the cable mount on the frame side [9].

Flat laboratory test tracks are subject to certain limitations, the most significant of which is the restricted length of the available measurement zone. Conversely, using drums instead of flat surfaces introduces other significant drawbacks, such as differences in tire deformation patterns on drums versus flat roads [4]. With few exceptions, laboratory tests are carried out on rigs equipped with rotating cylinders at least 2 m in diameter. In such cases, rolling resistance can be measured by detecting the force exerted at the hub of the test wheel as it rolls freely on the drum or by calculating the rolling resistance from the torque needed to rotate the drum shaft at a constant speed. Both the drum and hub bearings introduce parasitic resistance torques that interfere with measurements. To account for this, measurements are conducted under partially unloaded conditions to correct the results obtained under normal operating conditions.

The trailer method is not only used to determine rolling resistance, but has also been adapted to measure longitudinal and lateral forces acting on the wheel during turning maneuvers on different surfaces. In [10], a test rig is presented that allows the evaluation of wheel performance characteristics under various and specific ground conditions. Friction models describe the phenomena occurring at the contact surface between the tire and road. These models are built based on experimental results from laboratory or field studies. The most common friction models used in vehicle dynamics are the “magic formula” and the “LuGre” model [11,12,13,14,15].

The LuGre model (1) is one of the most widely used dynamic friction models in mechanics and robotics. It represents friction as a function of velocity and an internal state variable, while also accounting for the Stribeck effect [12]:

(1) $$\left\{\begin{array}{c} F=\left(\sigma_{0}z+\sigma_{1}\frac{dz}{dt}+\sigma_{2}v_{r}\right)F_{z}\\ \frac{dz}{dt}=v_{r}-\theta \frac{\sigma_{0}\left|v_{r}\right|}{g\left(v_{r}\right)}z\\ g\left(v_{r}\right)=\mu_{c}+\left(\mu_{s}-\mu_{c}\right)exp\left(-{\left|v_{r}/v_{s}\right|}^{\alpha_{s}}\right) \end{array}\right.$$

where $\sigma_{0}$ denotes the longitudinal elasticity coefficient of the bristle; $\sigma_{1}$ and $\sigma_{2}$ correspond to the longitudinal damping coefficient and the relative viscous damping coefficient, respectively; z represents the average deformation of the bristle; F refers to the frictional force and $F_{z}$ to the normal (vertical) load; and parameter θ characterizes the road surface condition. The relative velocity between contact surfaces is denoted $v_{r}$ and the function $g\left(v_{r}\right)$ accounts for the Stribeck effect. The friction coefficients $\mu_{c}$ and $\mu_{s}$ represent the Coulomb and static friction values. The symbol $v_{s}$ defines the Stribeck characteristic velocity, whereas $\alpha_{s}$ is the Stribeck exponent indicating steady-state friction behavior.

A widely used semiempirical tire friction model is the Pacejka model (2), commonly referred to as the “magic formula” [14,15]. The steady-state characteristics of the wheel’s longitudinal force and aligning moment are expressed through a set of parameters described below:

(2) y(x)=D⋅sin(C⋅arctan(Bx−E(Bx−arctan(Bx))))

where $y\left(x\right)$ refers to the output variable (typically longitudinal or lateral force or aligning torque), x denotes the slip parameter (e.g., slip ratio or slip angle), and B, C, D, E represents the curve fitting constants. This formulation enables accurate approximation of tire–road interaction forces under steady-state conditions and is widely implemented in vehicle dynamics modeling and simulation environments.

Rolling phenomena are also analyzed using numerical modeling. Both mesh-based and mesh-free methods are employed to simulate interactions at the wheel–surface interface [16,17]. For example, in [18], a dynamic model of an omnidirectional wheel was developed using the Modelica language. The model accounts for the transition of the contact point between rollers during rolling and introduces algorithms for contact tracking in two configurations: rollers aligned parallel to the wheel plane and rollers mounted at an angle. This approach ensures smooth, impact-free contact transfer and has been validated through numerical simulations. Nevertheless, experimental studies remain essential for model verification and parameter identification.

As previously outlined, the frictional interaction between the wheel and ground is a key component of vehicle dynamics research and is particularly relevant for improving mobile robot control [19]. In recent years, omnidirectional wheels—both Mecanum and standard omni-wheels—have become increasingly common in autonomously guided vehicles [20]. These wheels differ in their rolling surface from standard automotive tires. While various experimental methods exist for characterizing tire friction, there is a lack of studies focusing on the frictional forces in mobile robot wheels.

In [21], the LuGre model was applied to characterize the longitudinal friction of a generic omni-wheel. A custom test stand was developed to identify the LuGre model parameters for three different floor surfaces. Additionally, the study investigated—for the first time—the influence of lateral friction on longitudinal motion in omnidirectional wheels. The results indicated that the measured traction forces exhibited an oscillatory pattern. These oscillations are due to the specific design of omni-wheels, where surface contact occurs through successive transitions between passive rollers. This finding was corroborated by the authors of [22].

To mitigate discontinuities in wheel–ground contact, various studies have focused on optimizing the design of omnidirectional wheels [23,24], particularly by minimizing the gaps between adjacent rollers. These efforts aim to reduce contact discontinuities and consequently vibration generation. However, these improvements do not eliminate vibrations resulting from the wheel’s geometrically varied circumference. To address this, optimally tuned suspension systems are often used to isolate vibrations from omni-wheels and protect the vehicle chassis [25,26].

Omnidirectional wheels are equipped with passive rollers that enable motion both parallel and perpendicular to the wheel’s axis of rotation, significantly reducing lateral friction. These wheels lack a steering axis and are fixed directly to the vehicle frame. Therefore, to simulate a vehicle driving along a curved path, it is necessary to determine the motion resistance forces for such wheel kinematics.

This study presents a test stand and experimental results aimed at characterizing the motion resistance generated by an omnidirectional wheel during both straight-line and turning maneuvers. The developed test stand enabled the evaluation of motion resistance useful for modeling the dynamics of vehicles equipped with such wheels.

It should be emphasized that the literature contains virtually no studies showing how the rolling resistance changes with the steering angle of an omnidirectional wheel. In this respect, the data presented in this paper are novel and provide a reference for developing new modeling methods that incorporate the observed results—specifically, the resistance forces that can be expected during low-speed maneuvers. The objective of this study was therefore to provide reference data for calibration and further development of models for omnidirectional wheels.

## 2. Description of the Test Stand

To conduct studies aimed at determining motion resistance during the rolling of an omnidirectional wheel, a proprietary test stand—originally presented in [22]—was adapted through relevant modifications. The original stand was designed for measuring vibrations transmitted to a suspension column caused by wheel–ground contact, depending on both the shape of the wheel and the condition of the surface it rolls over. The test stand is protected by a patent in Poland.

**Figure 1:**
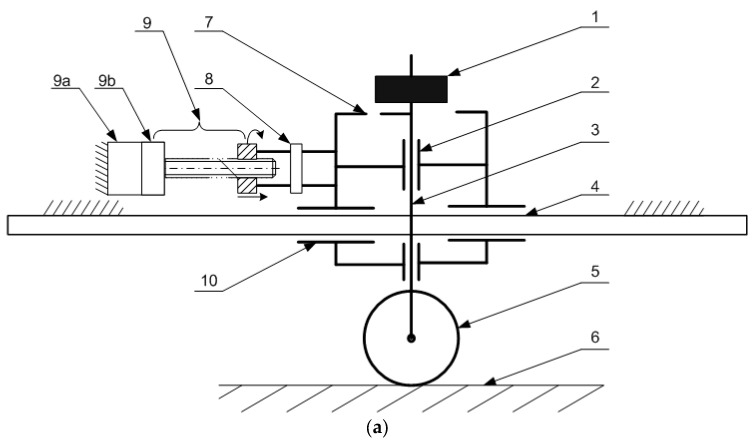

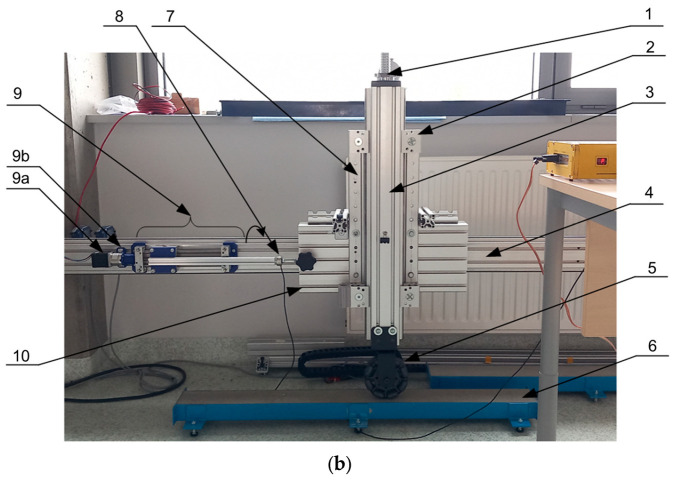
Structure of the test stand with numbered components: (**a**) schematic diagram of the device, (**b**) actual object.

The modifications involved altering the drive mechanism of the cart (10), which moves the column (3), to enable the installation of a force sensor (8) in the drive system. Figure 1 presents the proposed test stand: (a) schematic diagram of the setup and (b) photograph of the actual stand. Both illustrations include element numbering for clarity.

The operation of the proposed device is based on inducing motion of the vertical column (3) to which the omnidirectional wheel (5) is attached, using a linear drive (9) that actuates the cart (10). The wheel (5) moves over a horizontal surface (6), which can be made from a variety of materials, such as industrial concrete with different grain sizes or aluminum profiles. 

The linear drive (9) is powered by an electric motor (9a) with a gear ratio of 13.73:1 (9b), and is integrated with a control system that allows for precise control of the cart’s (10) movement speed, including acceleration and deceleration characteristics. The vertical column (3) is slidably connected to the cart (10), allowing it to move freely in the vertical direction in response to external forces, such as irregularities in the ground surface (6) or the wheel’s (5) envelope geometry. The column (3) can move vertically via a set of bearings (2) installed on the cart frame (7). The vertical displacement of the column (3) relative to the guide (4)—which serves as a reference frame—can be measured using displacement or acceleration sensors, although these were not used in the current study.

The wheel can be loaded as needed by applying additional weights (1). The force required to initiate motion of the omnidirectional wheel is measured using a force sensor (8).

The use of a linear force sensor is justified by several important technical and practical considerations. First, measuring longitudinal force aligns directly with the direction of rolling resistance forces. This means that the measured value corresponds directly to the force opposing the wheel’s displacement, eliminating the need to convert torque into force using additional geometric parameters.

Second, the linear method, with the sensor mounted directly in front of the cart (9), minimizes the influence of play and friction in the drive system components, which could distort results if torque on the drive shaft were measured instead.

Third, under quasi-static conditions (low speeds and absence of acceleration), measuring rolling resistance in a linear setup accurately reflects the forces occurring in the wheel’s actual operating environment. Torque becomes less useful in this case, as it does not fully represent the work done by the system in the direction of displacement.

Another advantage of this approach is its simplicity in construction and calibration: linear force sensors are more cost-effective, easier to implement, and allow the test stand to be easily adapted for different wheel types without requiring special adapters. Finally, this method eliminates additional computational transformations, significantly simplifying data analysis and enabling direct use of the measurements for calibrating numerical models of wheel–surface interaction.

An alternative method for determining resistance forces could involve measuring torque directly on the wheel. However, for omnidirectional wheels, this approach would introduce significant interpretation challenges. The torque recorded at the wheel hub would include the combined effects of passive rollers operating in various directions, rather than solely the longitudinal rolling resistance. As a result, it would be difficult to isolate the force component parallel to the direction of motion, which is critical for evaluating the energy efficiency of the drive system. Furthermore, torque measurement would require special shafts, couplings, and bearings, introducing additional sources of friction and complexity to the measurement setup. For different wheel types, custom adapters for torque sensors would also be necessary, reducing flexibility and increasing testing costs.

Therefore, the adopted method for measuring the rolling resistance of an omnidirectional wheel assumes that the total measured tractive force consists not only of the rolling resistance components but also of other resistance forces, which must be taken into account in the calculations:

(3) $$F_{c}=F_{r}+F_{f}+F_{b}$$

where *F_r_*—rolling resistance of the trailing wheel [N], *F_f_*—resistance in the guide frame [N], and *F_b_*—frictional resistance force in bearings [N].

The force *F_f_* resulting from friction between the support beam (4) and the cart frame (10) was determined experimentally by moving the cart with the wheel lifted off the ground. To ensure the repeatability and reliability of the results, all components that could generate additional friction, such as roller end caps with felt wipers, were removed from the test stand. The guide rails and rollers were thoroughly cleaned of any wear residue and subsequently lubricated in accordance with the manufacturer’s recommendations. The measured resistance force was approximately 20% of the total resistance observed during wheel rolling. As shown in [27], resistance in the bearings *F_b_* is significantly smaller than the wheel’s rolling resistance. Therefore, it was decided not to determine this component separately, especially considering that the test wheel and its bearings constitute a single integrated unit. Consequently, in any design application, the motion resistance of the omnidirectional wheel will be the sum of the rolling resistance from contact with the surface and bearing friction, both contributing, albeit to different degrees, to the total resistance force *F_c_* measured in the experiments.

The constructed test stand allows measurements to be performed on wheels of various types and designs. In this study, the focus was on a double-row omnidirectional wheel, with a detailed description of the selected model provided in Section 4.

## 3. Drive and Measurement System

**Figure 2:**
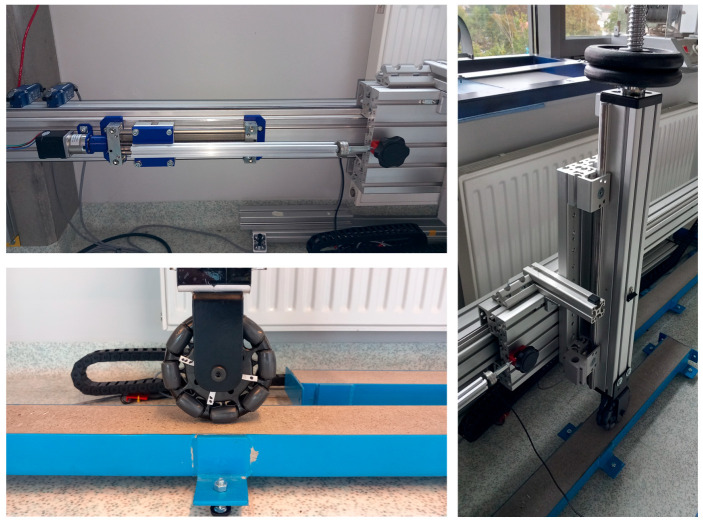
Research stand.

The drive system was constructed using standard components from the Item company. The electromechanical part of the test stand (Figure 2) consisted of the following elements: a Drylin linear drive module with preloaded tension, model SLWE-1040-PL from Igus, equipped with a trapezoidal lead screw with a pitch of 2 mm/rev. A bipolar stepper motor Nema 17, model 17HS19-1684S-PG14, was mounted at one end of the screw.

A linkage was attached to the linear drive cart, with the opposite end connected to the cart of the mechanism that moves the column equipped with the omnidirectional wheel. A force sensor, EMS20-100 N from PPH WOBIT E.K.J., was installed within this linkage to measure the force required to move the tested wheel, representing the resistance generated during wheel rolling.

**Figure 3:**
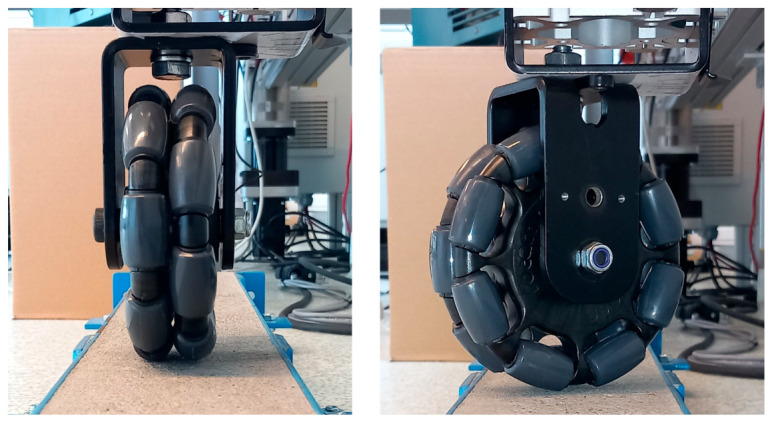
Example angular orientations of the omnidirectional wheel during testing.

The mounting of the omnidirectional wheel to the vertical column allows it to be oriented at various angles relative to the direction of cart movement in a range from 0° to 90° (Figure 3). This makes it possible to simulate wheel contact in straight-line motion as well as in curved trajectories.

**Figure 4:**
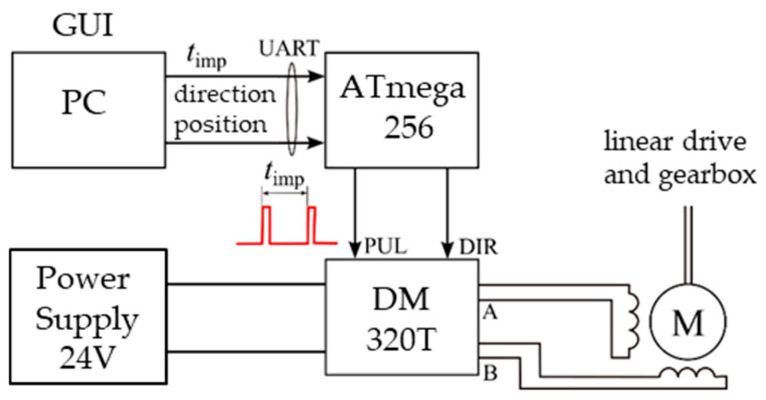
Diagram of the cart speed control system.

The cart’s speed is controlled by regulating the rotational speed of the stepper motor that drives the lead screw. The control system used during the experiment is shown schematically in Figure 4.

The stepper motor itself is powered by using a DM320T driver, which is compatible with Nema 17 motors. During testing, the driver was set to full-step mode and powered by a 24 V DC power supply. The stepper motor operated in an open-loop configuration (without feedback), with its rotational speed regulated by controlling the time interval t_imp_ between pulses sent to the PUL input, where each pulse represents a single step [28].

The use of a gear and lead screw enabled low-vibration movement, despite the discrete nature of the motor’s operation. The direction of cart movement was defined by the rotation direction of the stepper motor, determined by the signal sent to the DIR input of the driver. Both DIR and PUL signals were generated by an ATmega 2560 microcontroller. The signal characteristics, particularly the time between pulses (which directly determines the drive speed), were defined in a PC-based graphical user interface (GUI) and transmitted to the microcontroller via UART [29]. To ensure precise timing of step pulses (and therefore drive speed), pulse generation was handled by the ATmega’s hardware timer interrupt system. In addition to speed control (from which t_imp_ was calculated), the user interface allowed for setting bidirectional oscillatory motion between defined positions, as well as turning the drive system on or off.

The measurement system used during the experiments focused on force measurement. The EMS20 sensor from WOBIT, operating within the 0–50 N range, was used for this purpose. The analogue signal was recorded using a ESAM Traveler 1 data acquisition system. Real-time measurements were captured on a PC, and it was also possible to preview the sensor’s output during the experiment.

## 4. Experimental Research

The study concerning the determination of rolling resistance for an omnidirectional wheel was carried out under quasi-static conditions, at very low velocities. A classic support wheel from Rotacaster was used for the experiment. The wheel had a diameter of 125 mm and was equipped with a double row of passive rollers. The main specifications of the wheel are provided in Table 1.

**Figure 5:**
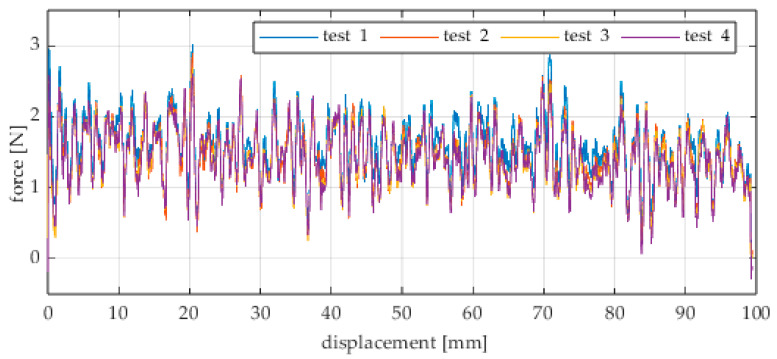
Friction force between the cart and the guide as a function of displacement.

The force measured by the sensor is composed of both the rolling resistance of the wheel and the friction occurring between the cart and the guide rail. Therefore, as a first step, these friction forces were identified by conducting a series of runs with the wheel removed. The results showed that the resistance forces were repeatable and dependent on the cart’s position (the repeatability of the friction force between the cart and the guide rails was a necessary condition to enable its subtraction from the total measured resistance force). The identified friction force values are shown in Figure 5 and were subtracted from the measured forces in subsequent experiments.

During the tests, the wheel was subjected to two different loading conditions:

- Only the weight of the column (resulting in a wheel load of 117.7 N).
- The column plus an additional mass of 10 kg (resulting in a wheel load of 215.8 N).

Measurements were conducted for five wheel orientation angles relative to the direction of movement: 0°, 30°, 45°, 60°, and 90°. This setup allowed for the simulation of different movements:

- In a straight line (0°).
- Along a curve (30°, 45°, 60°).
- Pivoting in place (90°), which corresponds to rotation around its own axis.

Due to the limited displacement range of the column (approximately 100 mm), four measurement runs were recorded for each angular position. The initial wheel position was changed by 90° for each run, i.e., measurements started from each quarter of the wheel’s circumference. These measurement series are labeled “quarter I–IV” in the plots. The movement speed of the column (5) was identical in all variants and was Vx = 1.67 mm/s (approximately 0.0017 m/s).

The tests were conducted at an ambient temperature of 22 °C, with a sampling frequency of 100 Hz. Measurements were carried out on two different surfaces: an industrial concrete floor and an aluminum profile. These two types of surfaces were selected because they are representative of industrial and laboratory environments. Concrete floors replicate conditions typically encountered by vehicles equipped with omnidirectional wheels, whereas anodized aluminum, due to its availability and uniform surface characteristics, provides controlled roughness along the measurement path. For the semi-finished aluminum product used in the tests, the surface roughness Ra over the measurement length was 0.32 µm, measured using a Taylor Hobson Surtronic 25 device.

The industrial concrete floor exhibited varying grain sizes, introducing some randomness in the wheel–surface contact area, which was difficult to characterize precisely. Nevertheless, using such a surface allows simulation of real operating conditions in which omnidirectional wheels are typically employed.

### 4.1. Experimental Tests on a Concrete Surface

The first series of tests was conducted with the wheel rolling over an industrial concrete floor. Figure 6, Figure 7, Figure 8, Figure 9, Figure 10, Figure 11, Figure 12, Figure 13, Figure 14, Figure 15 and Figure 16 present the rolling resistance measurements for various loading conditions and different angular orientations of the wheel relative to the direction of motion.

The measured force values were additionally presented with respect to the wheel’s rotation angle using Polarplot. The radius of the plot included an axis indicating the value of the resistance force. It should be noted that the greater the turning angle of the wheel around the vertical axis, the lower its angular velocity. The method used to measure these angles is described in [22]. The plots do not include the variant in which the plane of the wheel is set at an angle of 90° to the direction of column movement. For this configuration, the wheel’s self-rotation speed is zero, and it moves only via the passive rollers located along its circumference. Due to the greater variability of values in this case, the plots were prepared specifically for the runs performed with the additional load mass applied.

The characteristic values obtained from the tests (maximum, minimum, mean values, and standard deviation) are summarized in Table 2.

As expected and consistently with the current state of knowledge, the rolling resistance force increases with higher load. However, this change is not linearly proportional. Notably, the results reveal a clear influence of the wheel orientation angle on the magnitude of the resistance force in the longitudinal direction. It is significant that for both load cases, the highest average resistance force was recorded for the wheel oriented at 60° relative to the direction of travel. These effects may be associated with the presence of additional force components and the nonlinear deformation of the elements in contact with the surface.

Additionally, noticeable force oscillations occur, and their characteristics vary depending on the wheel orientation angle. This results from the fact that at higher steering angles, the entire wheel rotates significantly slower (as shown in polar plots), while the rotational motion of the rollers on the wheel circumference becomes more influential. Since these rollers have a smaller diameter, they rotate faster, resulting in greater variability of force.

Most of the recorded plots include negative force values, indicating a reversal in the force direction: the column with the wheel occasionally pushed against the drive screw with the sensor, rather than being pulled by it. This phenomenon occurs because the circumference of the omnidirectional wheel is not perfectly circular. Due to these irregularities, the wheel reached an unstable equilibrium at certain points and then rolled forward under the influence of gravity. Such irregularities on the wheel’s circumference may be caused by several factors, including manufacturing inaccuracies, assembly misalignments, material seams, or deformations. It was also observed that the force distribution was not symmetrical around the mean value, which may suggest that the vertical axis of the wheel was not perfectly perpendicular to the surface such that a slight inclination of the wheel axis could cause one row of rollers to carry higher loads.

### 4.2. Experimental Tests on an Aluminum Surface

Analogous measurements were conducted for an omnidirectional wheel rolling on an aluminum surface. In these tests, industrial concrete blocks were replaced by an aluminum profile. The obtained results are shown in Figure 17, Figure 18, Figure 19, Figure 20, Figure 21, Figure 22, Figure 23, Figure 24, Figure 25, Figure 26 and Figure 27.

The characteristic values obtained from the tests on the aluminum surface (maximum, minimum, mean values, and standard deviation) are summarized in Table 3.

Similarly to the concrete surface tests, the results confirm that rolling resistance increases with higher load, although the relationship is not linear. The wheel orientation angle has the most significant influence on the resistance force. For both load levels, the highest mean resistance value was recorded at 60°, which suggests the presence of additional force components related to roller engagement.

As in the previous series, an oscillatory force pattern was observed, becoming more pronounced at higher steering angles. This occurs because at large angles, the entire wheel rotates more slowly while individual rollers move faster along the surface, increasing the variability in the contact force.

Negative force values observed in many plots indicate moments when the test column pushed the drive screw rather than being pulled by it. As before, this phenomenon can be attributed to irregularities in the wheel circumference and temporary unstable equilibrium points. These irregularities may result from manufacturing inaccuracies, assembly clearances, or a slight tilt of the wheel axis, causing asymmetry in load distribution across the roller rows.

## 5. Summary and Conclusions

This paper presents the results of an experimental analysis of rolling resistance in omnidirectional wheels on two types of surfaces (concrete and aluminum) under quasi-static conditions. Tests were carried out for five wheel orientation angles (0°, 30°, 45°, 60°, and 90°) and two loads (117.7 N and 215.8 N). Based on the obtained results, the following scientific conclusions can be formulated.

- Rolling resistance increases with increasing load; however, this relationship is not linear, which may result from nonlinear deformations in the contact zone.
- Wheel orientation has a key influence on rolling resistance: the highest average values were recorded at an angle of 60°.
- The recorded force profiles exhibit an oscillatory nature, associated with the transition of contact between successive rollers of the wheel.
- The occurrence of negative force values results from irregularities in the wheel’s circumference and local points of unstable equilibrium.
- The type of surface (concrete vs. aluminum) had a smaller effect on the measured resistance forces than the wheel angle and load.

The conducted tests are subject to certain limitations. The current study focused on forces along the direction of motion. The lack of lateral force measurements limits the analysis of turning and curved trajectories. The tests were performed under quasi-static conditions, and results of such tests have not been published previously. The results presented in this paper highlight the challenge of applying commonly known wheel–surface contact models to quasi-static motion of omnidirectional wheels. This issue is challenging due to the discontinuous contact and oscillatory nature of the resistance force observed in this study. The data presented in this article, covering the variability of resistance force as a function of wheel orientation and load, constitute an important starting point for identifying and modifying the parameters of such models. Measuring forces as a function of velocity would allow calibration of models that account for velocity effects (e.g., the LuGre model), although this would also involve the influence of inertial effects.

Further development of the test stand should include a detailed analysis of wheel geometry and assembly accuracy to assess the impact of imperfections on force oscillations. It would also be desirable to extend the measurement section and upgrade the measurement system to enable determination of resistance forces in the lateral direction relative to motion.

The measurement results presented in this paper are valuable and can serve as input data for modeling wheel–surface interactions in omnidirectional wheels. They also constitute a dataset that can be used as a reference in further work on modeling omnidirectional wheels.

## Figures and Tables

**Figure 6:**
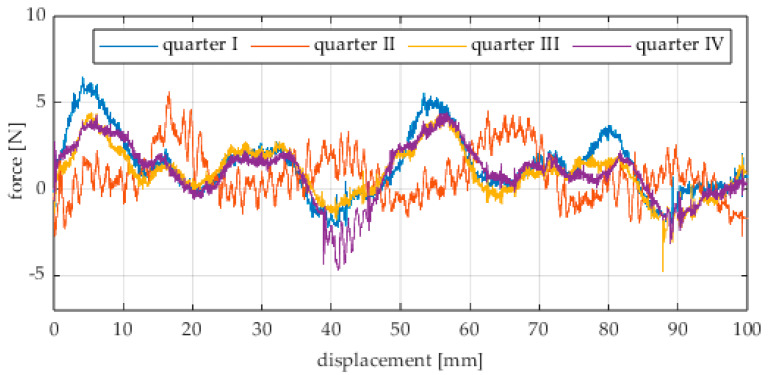
Rolling resistance force as a function of displacement for a load of 117.7 N and a turning angle of 0° for movement on a concrete surface.

**Figure 7:**
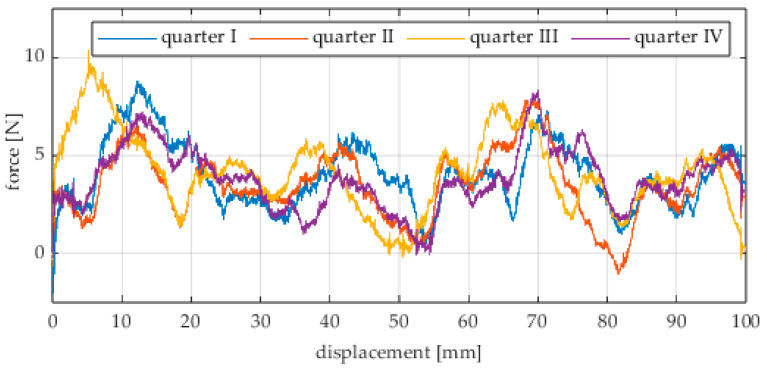
Rolling resistance force as a function of displacement for a load of 117.7 N and a turning angle of 30° for movement on a concrete surface.

**Figure 8:**
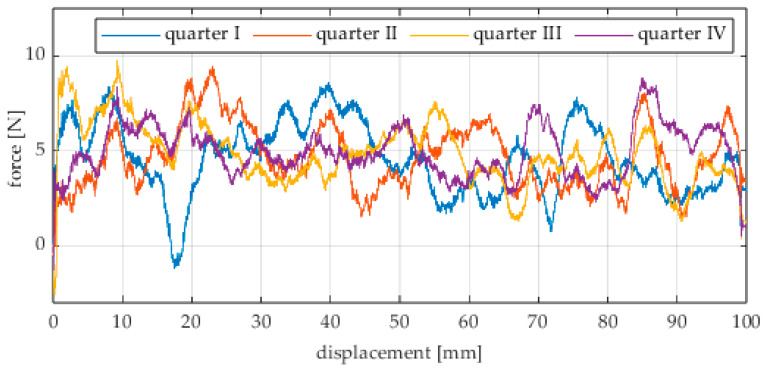
Rolling resistance force as a function of displacement for a load of 117.7 N and a turning angle of 45° for movement on a concrete surface.

**Figure 9:**
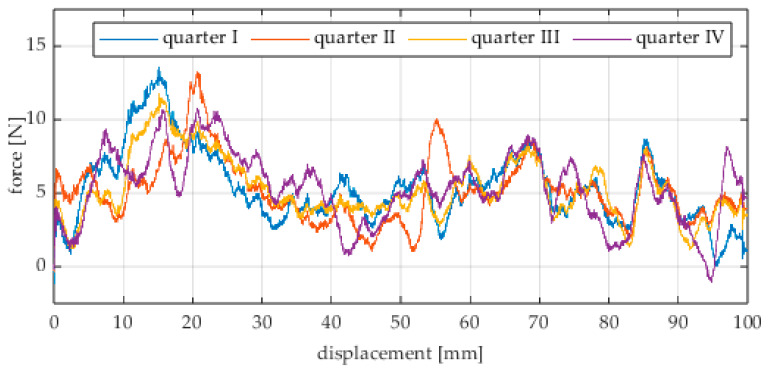
Rolling resistance force as a function of displacement for a load of 117.7 N and a turning angle of 60° for movement on a concrete surface.

**Figure 10:**
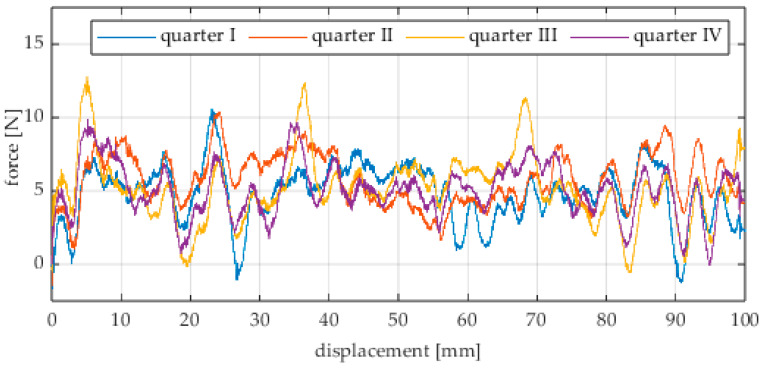
Rolling resistance force as a function of displacement for a load of 117.7 N and a turning angle of 90° for movement on a concrete surface.

**Figure 11:**
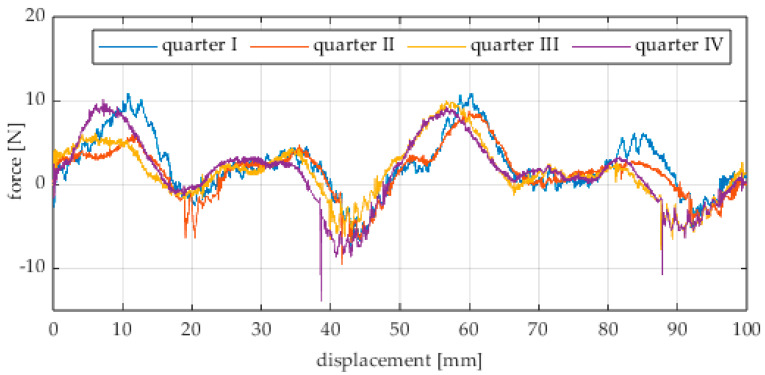
Rolling resistance force as a function of displacement for a load of 215.8 N and a turning angle of 0° for movement on a concrete surface.

**Figure 12:**
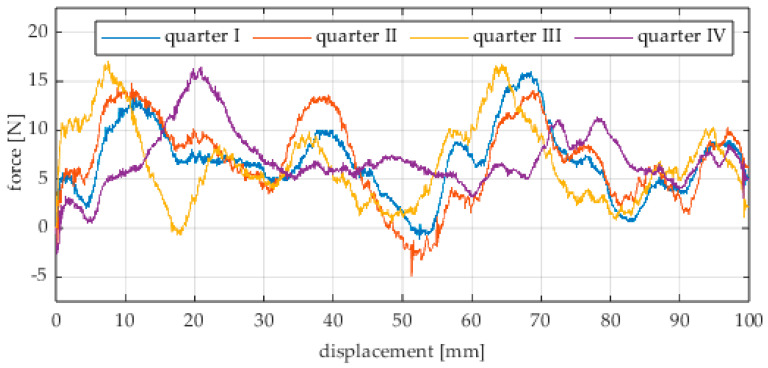
Rolling resistance force as a function of displacement for a load of 215.8 N and a turning angle of 30° for movement on a concrete surface.

**Figure 13:**
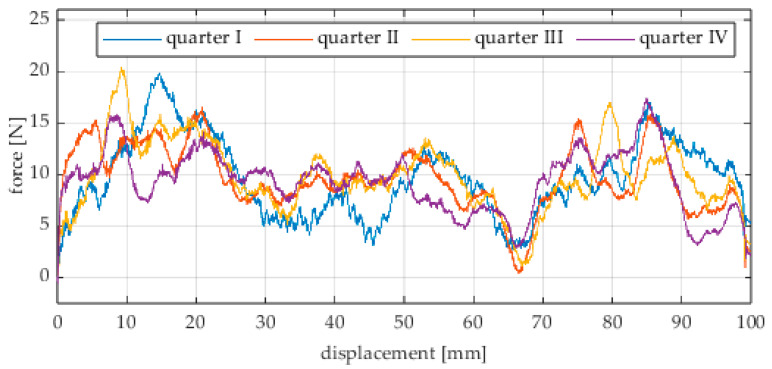
Rolling resistance force as a function of displacement for a load of 215.8 N and a turning angle of 45° for movement on a concrete surface.

**Figure 14:**
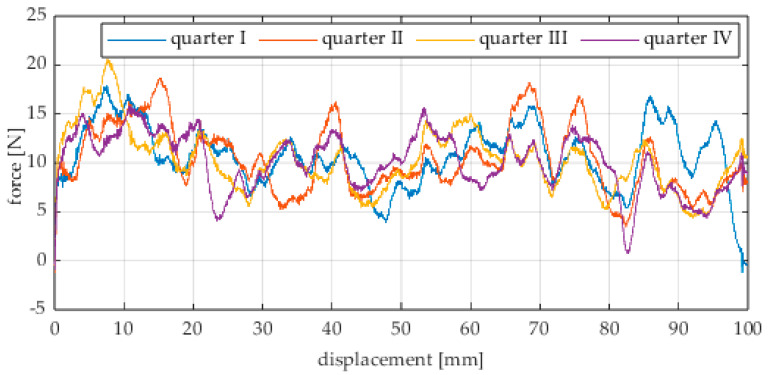
Rolling resistance force as a function of displacement for a load of 215.8 N and a turning angle of 60° for movement on a concrete surface.

**Figure 15:**
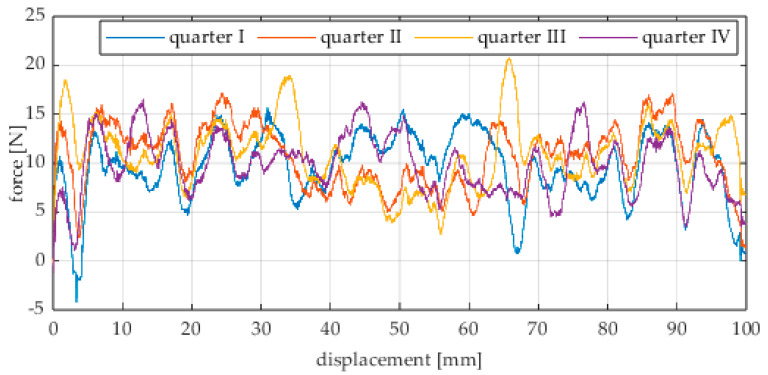
Rolling resistance force as a function of displacement for a load of 215.8 N and a turning angle of 90° for movement on a concrete surface.

**Figure 16:**
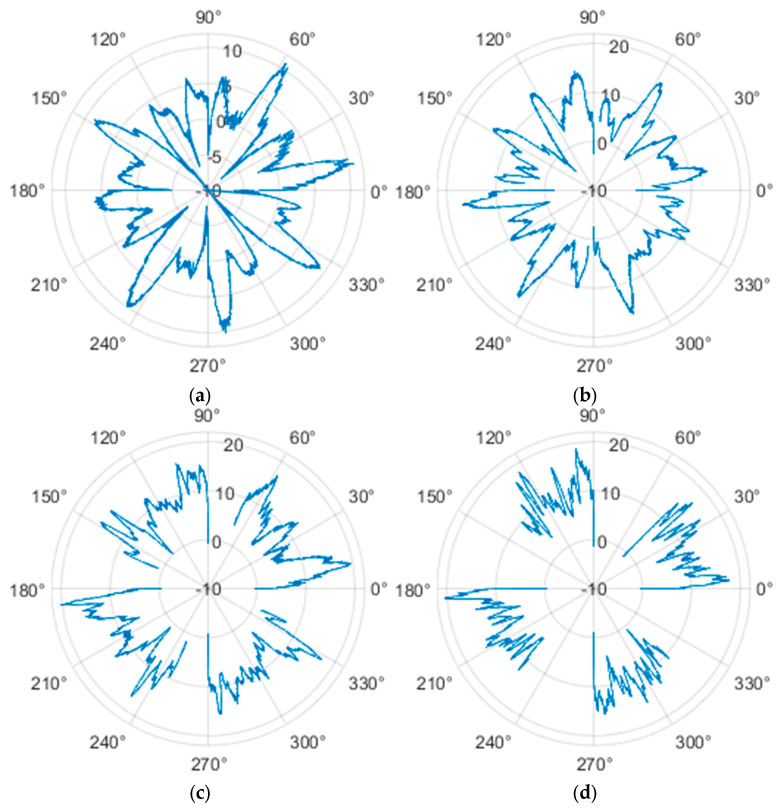
Rolling resistance forces of the omnidirectional wheel over 100 mm measurement segments along the wheel circumference, determined for motion on a concrete surface and for wheel turning angles of: (**a**) 0°, (**b**) 30°, (**c**) 45°, and (**d**) 60°.

**Figure 17:**
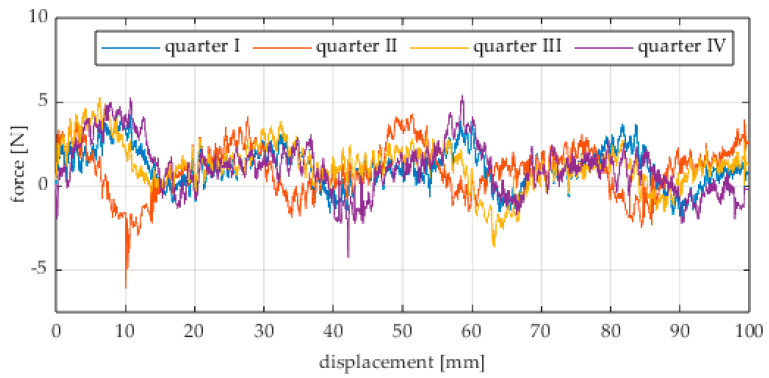
Rolling resistance force as a function of displacement for a load of 117.7 N and a turning angle of 0° for movement on an aluminum surface.

**Figure 18:**
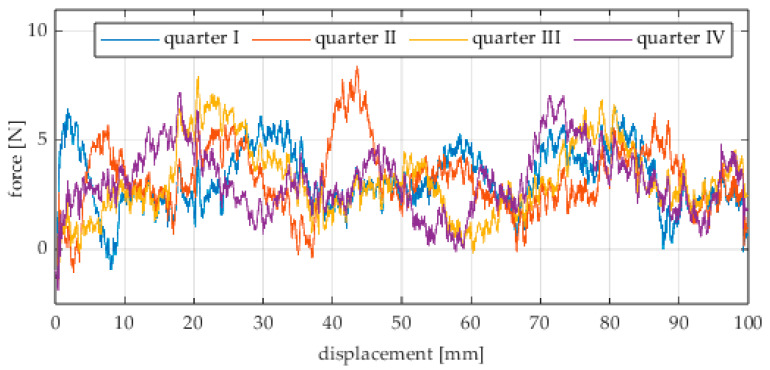
Rolling resistance force as a function of displacement for a load of 117.7 N and a turning angle of 30° for movement on an aluminum surface.

**Figure 19:**
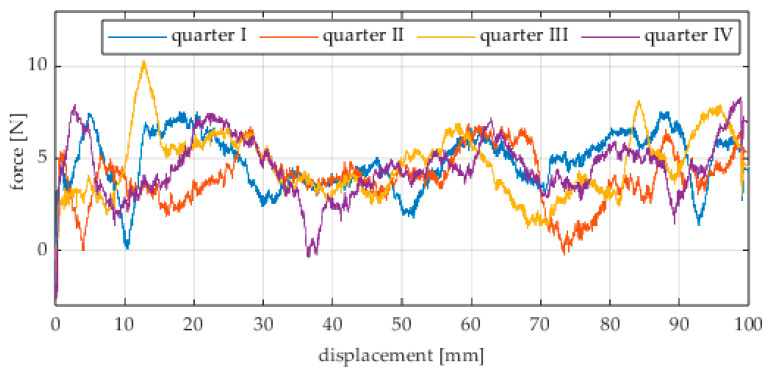
Rolling resistance force as a function of displacement for a load of 117.7 N and a turning angle of 45° for movement on an aluminum surface.

**Figure 20:**
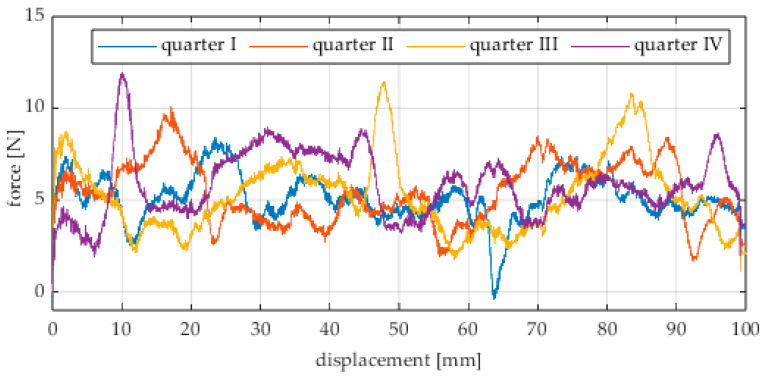
Rolling resistance force as a function of displacement for a load of 117.7 N and a turning angle of 60° for movement on an aluminum surface.

**Figure 21:**
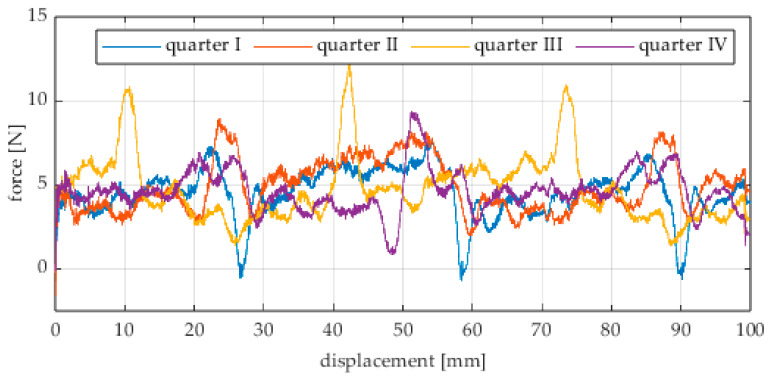
Rolling resistance force as a function of displacement for a load of 117.7 N and a turning angle of 90° for movement on an aluminum surface.

**Figure 22:**
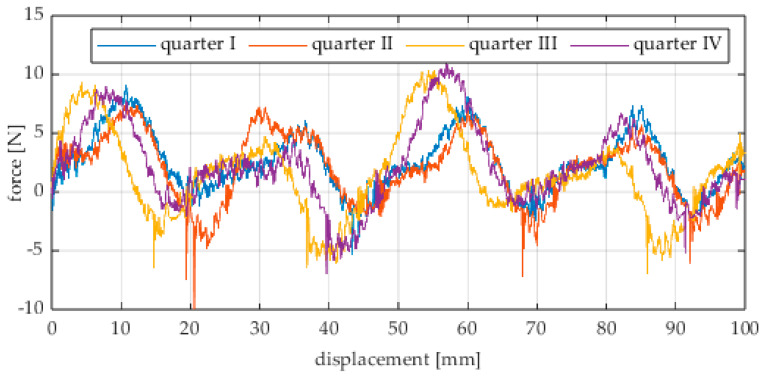
Rolling resistance force as a function of displacement for a load of 215.8 N and a turning angle of 0° for movement on an aluminum surface.

**Figure 23:**
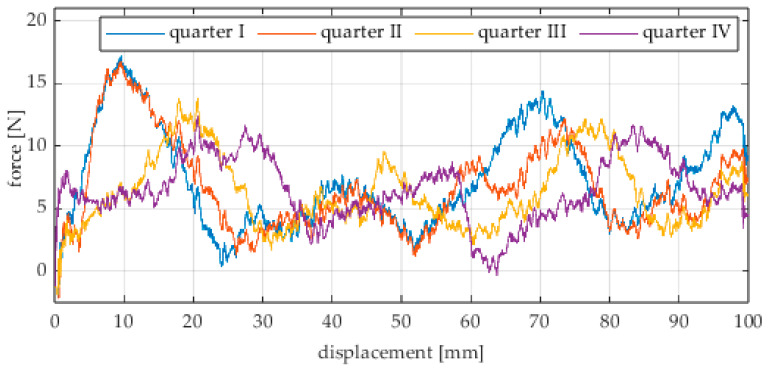
Rolling resistance force as a function of displacement for a load of 215.8 N and a turning angle of 30° for movement on an aluminum surface.

**Figure 24:**
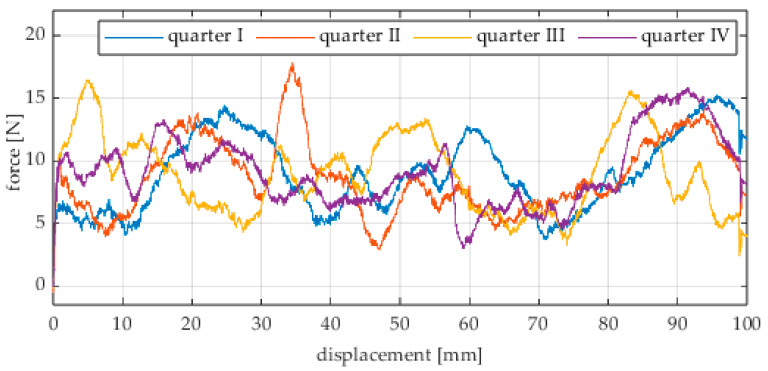
Rolling resistance force as a function of displacement for a load of 215.8 N and a turning angle of 45° for movement on an aluminum surface.

**Figure 25:**
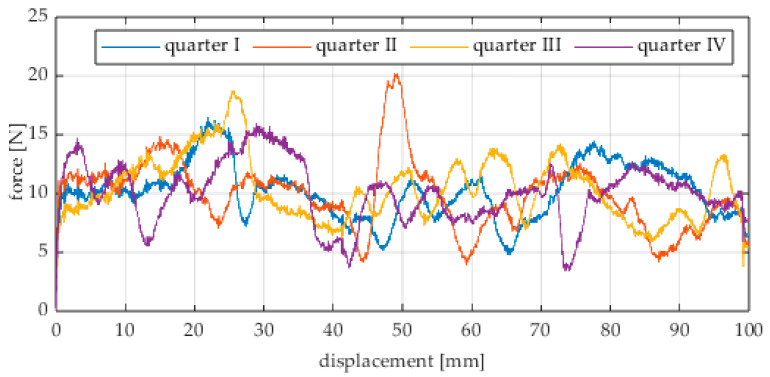
Rolling resistance force as a function of displacement for a load of 215.8 N and a turning angle of 60° for movement on an aluminum surface.

**Figure 26:**
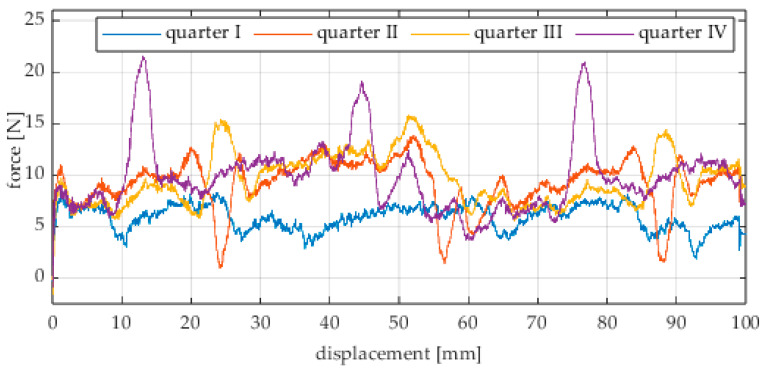
Rolling resistance force as a function of displacement for a load of 215.8 N and a turning angle of 90° for movement on an aluminum surface.

**Figure 27:**
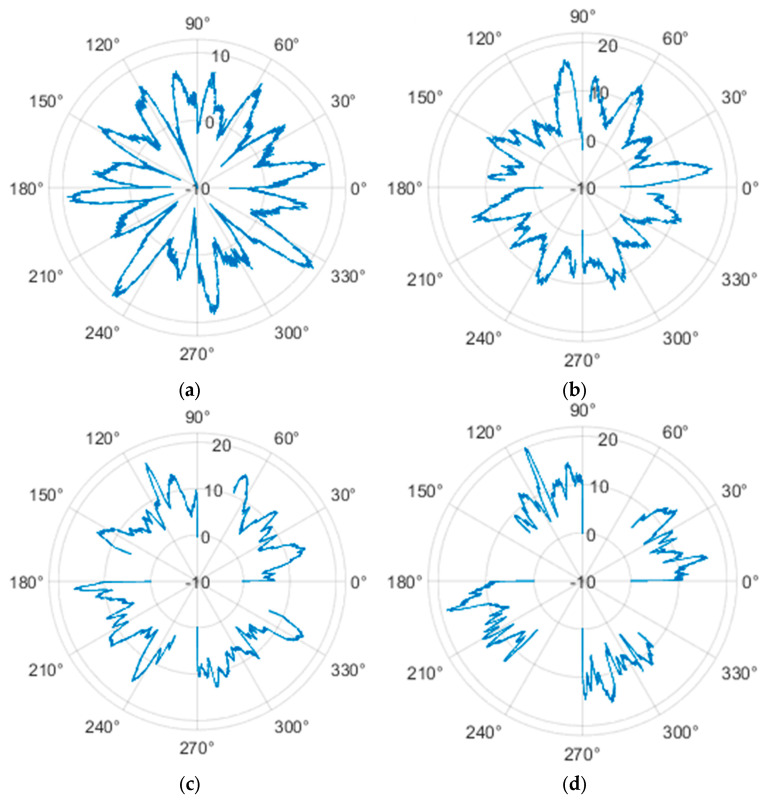
Rolling resistance forces of the omnidirectional wheel over 100 mm measurement segments along the wheel circumference, determined for motion on an aluminum surface and for wheel turning angles of: (**a**) 0°, (**b**) 30°, (**c**) 45°, and (**d**) 60°.

**Table 1:** Specifications of the Rotacaster 125 mm omnidirectional wheel used.

Parameter	Value	Unit
Catalogue number	R2-1258-95/S10	-
Outer diameter	125	mm
Width	43	mm
Number of passive rollers	8	-
Maximum roller diameter	20	mm
Roller material	Polyurethane	-
Roller hardness	95	Shorea
Main bearings	2 × ball bearing	-
Roller bearings	2 × Nylon sleeves per roller	-
Distance between roller rows	19	mm
Nominal distance between body and roller contact with surface	2.5	mm
Minimum distance between body and roller contact with surface	0.5	mm
Weight	0.315	kg

**Table 2:** Statistical summary of rolling resistance forces for different wheel angular orientations and loads on a concrete surface.

Wheel Angular Orientation [°]	Max. Force [N]	Min. Force [N]	Mean Force [N]	Standard Deviation [N]	Load [N]
0	6.507	−4.742	1.040	1.597	117.8
30	10.337	−2.036	3.838	1.763	117.8
45	9.784	−2.630	4.761	1.675	117.8
60	13.627	−1.159	5.289	2.300	117.8
90	12.819	−1.667	5.194	2.013	117.8
0	10.939	−13.843	1.669	3.504	215.8
30	17.060	−4.999	6.855	3.737	215.8
45	20.333	−0.672	9.602	3.362	215.8
60	20.612	−1.256	10.339	3.184	215.8
90	20.779	−4.153	10.249	3.324	215.8

**Table 3:** Statistical summary of rolling resistance forces for different wheel angular orientations and loads on an aluminum surface.

Wheel Angular Orientation [°]	Max. Force [N]	Min. Force [N]	Mean Force [N]	Standard Deviation [N]	Load [N]
0	5.414	−6.029	1.083	1.390	117.8
30	8.418	−1.921	3.165	1.532	117.8
45	10.375	−2.630	4.479	1.613	117.8
60	11.907	−0.428	5.398	1.734	117.8
90	12.191	−1.601	4.699	1.592	117.8
0	10.980	−10.179	2.066	3.177	215.8
30	17.168	−2.171	6.686	3.299	215.8
45	17.848	−0.537	8.939	3.009	215.8
60	20.289	−0.244	10.106	2.642	215.8
90	21.548	−1.601	8.596	2.962	215.8

## Data Availability

The original data presented in this study are included in the article. Further inquiries can be directed to the corresponding author.
